# Identification of Zika Virus NS1-Derived Peptides with Potential Applications in Serological Tests

**DOI:** 10.3390/v15030654

**Published:** 2023-02-28

**Authors:** Carlos Roberto Prudencio, Vivaldo Gomes da Costa, Leticia Barboza Rocha, Hernan Hermes Monteiro da Costa, Diego José Belato Orts, Felipe Rocha da Silva Santos, Paula Rahal, Nikolas Alexander Borsato Lino, Pâmela Jóyce Previdelli da Conceição, Cintia Bittar, Rafael Rahal Guaragna Machado, Edison Luiz Durigon, João Pessoa Araujo, Juliana Moutinho Polatto, Miriam Aparecida da Silva, Joyce Araújo de Oliveira, Thais Mitsunari, Lennon Ramos Pereira, Robert Andreata-Santos, Luís Carlos de Souza Ferreira, Daniela Luz, Roxane Maria Fontes Piazza

**Affiliations:** 1 Laboratório de Imunobiotecnologia, Centro de Imunologia, Instituto Adolfo Lutz, Av. Dr. Arnaldo, 351, São Paulo 01246-902, SP, Brazil; 2 Instituto de Biociências Letras e Ciências Exatas, Universidade Estadual Paulista Júlio de Mesquita Filho, Rua Cristóvão Colombo, 2265, São Jose do Rio Preto 15054-000, SP, Brazil; 3 Laboratório de Bacteriologia, Instituto Butantan, Av. Vital Brazil, 1500, São Paulo 05503-900, SP, Brazil; 4 Departamento de Microbiologia, Instituto de Ciências Biomédicas, Universidade de São Paulo, São Paulo 05508-000, SP, Brazil; 5 Instituto de Biotecnologia, Universidade Estadual Paulista Júlio de Mesquita Filho, Botucatu 18607-440, SP, Brazil; 6 Plataforma Científica Pasteur USP, Universidade de São Paulo, São Paulo 05508-000, SP, Brazil

**Keywords:** Zika virus, NS1 protein, monoclonal antibody, epitopes, peptides, immunoassays, diagnosis

## Abstract

Zika virus (ZIKV), a mosquito-borne pathogen, is an emerging arbovirus associated with sporadic symptomatic cases of great medical concern, particularly among pregnant women and newborns affected with neurological disorders. Serological diagnosis of ZIKV infection is still an unmet challenge due to the co-circulation of the dengue virus, which shares extensive sequence conservation of structural proteins leading to the generation of cross-reactive antibodies. In this study, we aimed to obtain tools for the development of improved serological tests for the detection of ZIKV infection. Polyclonal sera (pAb) and a monoclonal antibody (mAb 2F2) against a recombinant form of the ZIKV nonstructural protein 1 (NS1) allowed the identification of linear peptide epitopes of the NS1 protein. Based on these findings, six chemically synthesized peptides were tested both in dot blot and ELISA assays using convalescent sera collected from ZIKV-infected patients. Two of these peptides specifically detected the presence of ZIKV antibodies and proved to be candidates for the detection of ZIKV-infected subjects. The availability of these tools opens perspectives for the development of NS1-based serological tests with enhanced sensitivity regarding other flaviviruses.

## 1. Introduction

Zika virus (ZIKV), which belongs to the *Flaviviridae* family, is an emerging arthropod-borne flavivirus that has caused great concern, particularly in the Americas, since the last outbreak in 2015 [1]. ZIKV infection during pregnancy can lead to congenital Zika syndrome (CZS), among other birth defects, and has also been associated with Guillain-Barré syndrome in adults [2]. Currently, no vaccine or therapeutic agent against ZIKV is available, and controlling the disease is a challenge, especially with the co-circulation of other arboviruses [3]. Maintaining a case count is difficult because symptoms of the disease tend to be mild, and not everyone infected is seen by health services. Because these mild symptoms of ZIKV infections are similar to other flaviviruses diseases, many cases may not have been recognized [4,5]. A total of 7452 cases of ZIKV were reported, according to the World Health Organization (WHO), in 2020 [6]. Unfortunately, the ZIKV infection has recently resurged in India with the potential for devastating effects [6,7]. In Brazil, the possibility of the reemergence of the ZIKV epidemic has gained more strength due to the circulation of a new lineage, which serves as a warning to reinforce disease surveillance and diagnosis [8]. According to the Ministry of Health, between 2015 and 2022, 20,874 suspected cases of CZS were reported, of which 3707 (17.7%) were confirmed for some congenital infection. Guillain-Barré syndrome related to ZIKV infection reports on a total of 601 patients [9]. Up to week 48 of 2022, there were 9204 probable cases [10].

The potential spread of flavivirus infections has highlighted the need to improve disease diagnosis and monitoring methods [11]. Current diagnostic tests are focused on molecular or serological assays [12]. Molecular assays depend on direct molecular tests for the detection of viral nucleic acid in serum or blood up to seven days after the onset of symptoms or in urine up to 14 days post-infection, while immunoassays detect ZIKV-specific convalescent humoral immune response. Thus, both are highly dependent on the stage of infection [12]. The improvement of new antigenic tests would provide the fastest and cheap diagnosis during the acute phase, in which virus transmission is occurring, while the serological test, if highly sensible and specific, could give insight into previous infections or immunizations [13]. However, flavivirus serological methods are known to commonly be cross-reactive among different viruses [12].

ZIKV has a structure very similar to other Flaviviruses, mainly Dengue virus (DENV), West Nile virus (WNV), and Japanese encephalitis virus (JEV) [14,15]. The structural proteins (C, prM, and E) are used to assemble new virus shells, whereas the seven non-structural proteins (NS1, NS2A/B, NS3, NS4A/B, and NS5) are involved in various functions as in the replication of the viral RNA genome [16]. ZIKV-NS1 has a high biological relevance and, due to its immunogenicity, is an attractive target for drug development [17,18]. NS1 is secreted as a glycosylated hexamer in high amounts during the viremic phase [19]. The recombinant NS1 glycoprotein is extensively useful for immunological applications, such as serodiagnosis of ZIKV infection, and for monitoring immune responses based on IgM and IgG [20]. Nonetheless, the structural similarity between proteins encoded by these viruses hinders the development of sensitive and specific serological tests [21,22]. Indeed, serological assays based on NS1 protein have been used to detect previous infections by different flavivirus infections [20]. However, despite significant progress made in understanding ZIKV detection methods, further improvements are still needed [23,24].

Considering the relevance of specific and sensitive diagnostic methods for ZIKV infection, we used a purified recombinant form of the ZIKV-NS1 protein to immunize mice and rabbits to generate specific polyclonal (pAb) and monoclonal (mAb) antibodies to identify and characterize NS1-derived peptide epitopes. The present results demonstrate that some of these peptides are potential candidates for the development of ZIKV serological tests.

## 2. Materials and Methods

### 2.1. Ethics Statement

This study was conducted in accordance with the recommendations of Ethical Principles in Animal Research, adopted by the Brazilian College of Animal Experimentation. The protocol was approved by the Ethical Committee for Animal Research of Butantan Institute (6166050216 and 6265040216) and Adolfo Lutz Institute (CEUA/IAL no. 01/2022). All methods were performed in accordance with relevant guidelines and regulations and the ethical principles in animal research adopted using the ARRIVE 2.0 guidelines [25]. The Adolfo Lutz Institutional Human Research Ethics Committee (CEP/IAL, CAAE no. 97331218.7.0000.0059) approved the use of human serum samples. A free and informed consent form was obtained from the research subjects, and when it was impossible to obtain the signature, the confidentiality term was used for the remaining samples sent to the laboratory for routine examinations. For the use of serum samples for ELISA, the study was approved by the Research Ethics Committee of the São Paulo State University, Institute of Biosciences, Letters and Exact Sciences of São José do Rio Preto–SP (CAAE: 60482022.6.0000.5466). Positive and negative reference sera for ZIKV were composed of outpatients that attended the Emergency Care Unit in the city of Mirassol and the serum library of the Clinical and Molecular Virology laboratory, São Paulo.

### 2.2. Expression and Purification of the rZIKV-NS1 Protein

A recombinant plasmid (GenScript, Piscataway, NJ, USA), derived from pET-28a, encoding the ZIKV-NS1 protein (corresponding to the last 100 amino acids of the C-terminal region of ZIKV-NS1—patent application number: BR 10 2016 011,318 0)—was used to express the referred protein in *Escherichia coli* (*E. coli*), followed by purification using two chromatography steps according to an adapted protocol [26]. Briefly, the ZIKV-NS1 was expressed in *E. coli* Arctic Express (DE3) strain with 0.5 mM IPTG (Sigma Aldrich, St. Louis, MO, USA) for 18 h (18 °C, 200 rpm). The soluble protein fraction obtained after cell lysis was purified by affinity chromatography using a nickel-containing resin (Hiprep FF 5 mL, GE Healthcare Life Sciences, Chicago, IL, USA) in an ÄKTA–AVANT device (GE Healthcare Life Sciences). Samples containing the recombinant proteins were submitted to a second chromatographic step based on size exclusion chromatography using a Hiprep-SephacrylS-200HR 26/60 column (GE Healthcare Life Sciences). The purified ZIKV-NS1 was submitted to a final concentration step using nickel affinity chromatography based on the same experimental conditions applied in the first purification step. Finally, the purified ZIKV-NS1 was sorted in 15% polyacrylamide gels, quantified by means of image software (Image Lab, Build 16, Version 4.1, Bio-Rad, Hercules, CA, USA), and stored at −20 °C for subsequent uses.

The obtained ZIKV-NS1 was further used as an antigen for monoclonal and polyclonal antibody generation and characterization. The DENV2-NS1 protein was produced according to a described protocol [27] and employed as a control to evaluate the DENV cross-reactivity [28].

### 2.3. Production of Antibodies against the ZIKV-NS1 Protein

#### 2.3.1. Rabbit Polyclonal Antibody (pAb)

Two-month-old female New Zealand rabbits were immunized intramuscularly with 100-μg NS1-ZIKV adsorbed to the Freund complete adjuvant followed by one booster injection of the same antigen concentration with Freund incomplete adjuvant after 15 days intervals. Serum samples were obtained 15 days after the last antigen injection. Pre-immune serum samples were obtained just before first immunization by auricular venipuncture immunization to be used as negative controls in specific antibody evaluation. The reactivity of the immune sera was tested by ELISA [28]. Briefly, 96-well MaxiSorp microplates (Nunc^®^, Rochester, NY, USA) were coated for 2 h at 37 °C followed by another 18 h at 4 °C with a solution of 0.05 M, pH 9.6, sodium carbonate and bicarbonate containing 1 μg/mL of NS1-ZIKV. After blocking (1% BSA in PBS) for 45 min at 37 °C, the microplates were incubated for 1 h at 37 °C with polyclonal antiserum diluted from 1:100 to 1:6400, followed by incubation with goat anti-rabbit peroxidase-conjugated antibodies (Zymed, San Francisco, CA, USA), diluted 1:5000, for 45 min at 37 °C. Between steps, the reaction was washed with 0.05 Tween 20 in PBS, and at each step, the volume added was 100 μL/well, except in the washing and blocking steps, when the volume was 200 μL/well. All samples were tested in duplicate unless otherwise noted.

ELISA was developed with O-phenylenediamine (OPD; Sigma Aldrich Co, St Louis, MO, USA) plus 0.5-μL/mL hydrogen peroxide in 0.05 M citrate-phosphate buffer, pH 5.0, in the dark at room temperature. The reactions were interrupted after 15 min by the addition of 50 μL of 1 M HCl. The absorbance was measured at 492 nm in a Multiskan EX ELISA reader (Labsystems, Milford, MA, USA). The serum sample was filtered (0.45 μm) and purified by protein A affinity chromatography (GE Healthcare Life Science). The pAb-IgG enriched fraction purity was observed in a 12% SDS-polyacrylamide gel electrophoresis (SDS-PAGE) staining with Coomassie blue R-250.

#### 2.3.2. Mouse Polyclonal Antibody (pAb)

Female BALB/c mice (6–8 weeks of age and approximately 25 g of weight) were housed in a Ventilife Mice Mini-Isolator (Alesco^®^, Monte Mor, SP, Brazil) in a temperature-controlled light cycle facility in a Ventilife Ventilated Mouse Rack (Alesco^®^), at 25 °C and given antibiotic-free food and water *ad libitum*. For immunization, two groups of five mice each received three doses intramuscularly: 80 µg of pCAGGS plasmid containing the full ZIKV-NS1 protein gene or PBS (control group). Plasmid DNA was diluted in sterile PBS with 50% (*v*/*v*) of complete (first dose) or incomplete (second and third doses) Freund adjuvant. Mice were immunized at 21-day intervals. Blood samples were collected via the submandibular vein on day 0 (pre-immune) and at a frequency of 21 days before each dose. Mice were euthanized three weeks after the third dose by deep anesthesia with ketamine/xylazine use (140 mg/kg and 10 mg/kg, respectively). The blood samples were centrifuged at 3000× *g* for 15 min at room temperature, and the serum containing the mouse pAb anti-ZIKV NS1 was stored at −20 °C.

### 2.4. Isolation of Anti-ZIKV NS1 Monoclonal Antibodies (mAb)

Four-to-six-week-old female BALB/c mice were immunized via the footpad route with 10μg ZIKV-NS1 adsorbed in the Freund complete adjuvant. The immunization protocols consisted of two booster injections of the ZIKV-NS1 and Freund incomplete adjuvant after 4 weeks and at 15 days intervals between boosters. The mouse with the highest antibody titer was boosted with 10 μg of ZIKV-NS1 five days prior to cell fusion. The popliteal lymph node cells were fused with SP2/O-Ag14 mouse myeloma cells (2:1) using polyethylene glycol 1500 (Sigma Aldrich, St Louis, MO, USA) [29] with modifications [30]. The supernatant fluids were screened for specific antibodies by indirect ELISA in which 100 μL of hybridoma supernatant was added to 96-well MaxiSorp microplates (Nunc^®^, Rochester, NY, USA) previously coated with 1 μg/mL of purified ZIKV-NS1 or DENV-NS1 to screen cultures for antibody production. Antibody-secreting cells were expanded and cloned at limiting dilution [30].

### 2.5. ZIKV-NS1 mAbs Characterization

Hybridoma supernatants were incubated with each of the anti-isotype (anti-IgG1, anti-IgG2a, anti-IgG2b, anti-IgG3, anti-IgA, and anti-IgM antibodies) previously coated at MaxiSorp microplates followed by incubation with horseradish peroxidase-conjugated rabbit anti-mouse-IgG + A + M (1:1000) (Zymed, San Francisco, CA, USA). The supernatants from selected clones were filtered (0.45 μm) and purified by protein G affinity chromatography (GE Healthcare Life Science, Freiburg, Germany). mAb purity was observed in a 12% SDS-PAGE staining with Coomassie blue R-250. The detection limit was established using ZIKV-NS1 concentrations from 1 μg/mL to 0.6 ng/mL coated on microplates, followed by incubation with 10 μg/mL or 2.5 μg/mL of different anti-ZIKV-NS1 mAbs and with goat anti-IgG mouse conjugated with horseradish peroxidase (HRP) (1:5000) (Invitrogen, Carlsbad, CA, USA).

### 2.6. Checkerboard Titration

A polystyrene plate MaxiSorp (Nunc^®^, Rochester, NY, USA) was coated with 0.05 M carbonate/bicarbonate buffer, pH 9.6, containing serial dilutions of pAb ZIKV-NS1 from 60 to 1.875 μg/mL for 16–18 h at 4 °C and 2 h 37 °C. The wells were blocked with 1% BSA in PBS for 45 min at 37 °C. Then, 1 μg/mL of ZIKV-NS1 was added and incubated for 1 h at 37 °C. The mAbs were added in serial dilutions concentrations from 60 μg/mL to 0.156 μg/mL and incubated for 1 h at 37 °C. The detection was performed with goat IgG anti-mouse conjugated to peroxidase (Invitrogen, Carlsbad, CA, USA) diluted 1:5000, and the reaction was developed with OPD (Sigma Aldrich). The absorbance was measured at 492 nm, and the reaction without ZIKV-NS1 was taken for background noise subtraction. Between each reaction step, washing was performed with a PBS buffer containing 0.05% Tween 20.

### 2.7. ELISA Assays

The optimum concentration of pAb and mAbs ZIKV-NS1 as coating antigens and secondary antibodies, respectively, were determined by checkerboard titration procedures. Briefly, 96-well, polystyrene plates MaxiSorp (Nunc^®^, Rochester, NY, USA) were coated with serial dilutions of pAb ZIKV-NS1 from 15 to 0.03 μg/mL in a 0.05 M bicarbonate/carbonate coating buffer (pH 9.6) for 16–18 h at 4 °C (overnight) and 2 h 37 °C. TBS buffer with 0.5% casein was used as the diluent and for the blocking step for 2 h. Afterward, diluted sera (1:10) was added to the wells (100 μL/well) and incubated at 37 °C for 2 h; then, mAbs (2F2; anti-ZIKV-NS1) were added in serial dilutions concentrations from 10 to 0.7 μg/mL and incubated for 1 h at 37 °C. Subsequently, the plates were incubated with 100 μL of goat anti-mouse IgG conjugated to peroxidase (Invitrogen) diluted 1:10,000, and the reaction was developed with 100 μL of One-Step 3,3′,5,5′-tetramethylbenzidine (One-Step TMB, Scienco Biotech, Lages, SC, Brazil) colorimetric substrate. Finally, the reaction was stopped by adding 50 μL of 1 M hydrochloric acid, and then absorbance was measured at 450 nm. The wash solution (TBS 1X with 0.05% Tween 20) was used in all phases except after the addition of TMB (Scienco Biotech) in the final phase. Regarding the panel of samples to be screened, the cut-off value was determined as the mean absorbance of the negative sera ± 3 standard deviations (SD). Herein, 61 sera samples collected from 2016 to 2020 were tested, from which 27 and 34 were previously characterized as positive or negative for the presence of ZIKV, respectively.

### 2.8. Epitope Characterization and Structure Analysis of pAb-ZIKV and mAb IgG2a-ZIKV (2F2)

Peptide mapping was performed using an array of PEPperPRINT (Heidelberg, Germany). The microarray contained eight NS1 proteins of ZIKV, DENV1, DENV2, DENV3, DENV4, YFV, WNV, and JEV were linked and elongated by neutral GSGSGSG linkers at the C- and N-terminal regions to avoid truncated peptides. The extended and linked antigen sequences were translated into 15 amino acid peptides with a peptide-peptide overlap of 14 amino acids. The resulting NS1 peptide microarrays contained 2997 different peptides printed in duplicate (5994 spots) and were framed by additional HA (YPYDVPDYAG, 80 spots) and c-Myc (EQKLISEEDL, 80 spots) control peptides.

The reaction was performed as follows: the slides were blocked with Rockland blocking buffer MB-070 (30 min before the first assay), followed by incubation with 1 μg/mL of mAb 2F2-ZIKV or 10 μg/mL of pAb-ZIKV in PBS, pH 7.4 with 0.05% Tween 20 containing 10% blocking buffer MB-070 for 16 h at 4 °C with shaking at 140 rpm. Next, the slides were incubated with Goat anti-mouse IgG (H + L) DyLight680 (0.2 µg/mL) or sheep anti-rabbit IgG (H + L) DyLight680 (0.2 µg/mL) for 45 min staining in incubation buffer at room temperature. Between steps, the slides were washed for 3 × 10 sec after each incubation step with PBS, pH 7.4 with 0.05% Tween 20. Mouse monoclonal anti-HA (12CA5) DyLight800 (0.5 µg/mL); 45 min staining in the incubation buffer at RT was employed as control. The reactions were read at LI-COR Odyssey Imaging System, scanning offset 0.65 mm, resolution 21 µm, scanning intensities of 7/7 (red = 680 nm/green = 800 nm). Pre-staining of two NS1 peptide microarray copies was performed either with the secondary goat anti-mouse IgG (H + L) DyLight680 antibody or with the secondary sheep anti-rabbit IgG (H + L) DyLight680 antibody and the control antibody in incubation buffer to investigate background interactions with the antigen-derived peptides that could interfere with the main assays.

### 2.9. Sequence and Structure Analysis

We employed the PyMol program (DeLano Scientific LLC, San Carlos, CA, USA) to predict the structure and the epitope of NS1. For the NS1 structure, we used the available PDB file from Protein Data Bank (code: 5K6K) [31]. For the structure of monoclonal antibodies, we first performed the prediction with Phyre [32]. Multiple Sequence Alignment was performed by CLUSTALW. In order to predict N-glycosylation and phosphorylation sites, the selected epitope sequences were submitted to NetNGlyc and NetPhos 3.1 software, respectively [33,34]. In addition, to predict linear B cell epitope regions and protective antigens, the ABCPred and VaxiJen servers were used [35,36].

### 2.10. Peptides Synthesis

Employing the results generated by the arrays, the following peptides were synthesized at Aminotech (Diadema, SP, Brazil) with 95% purity (Table 1). Lyophilized peptides were diluted at 1 mg/mL concentration in phosphate-buffered saline (PBS) pH 7.4, divided into 100 μL aliquots, and stored at −30 °C for further use.

### 2.11. NS1 Peptide Dot Blot Assay Screening

For dot blot assay, 15 human sera samples were used, of which five were ZIKV positive, five were DENV positive, both by RT-PCR, ELISA, and MAC-ELISA, and five were negative for both viruses by RT-PCR, ELISA, and MAC-ELISA. The optimum concentrations of all sera, ZIKV peptides (ZKvROX1-ZKvROX6) as coating antigens and secondary antibodies, respectively, were determined by checkerboard titration procedures. For dot blot assay using rabbit and mouse pAb and mouse mAb, high-binding 0.22 µm nitrocellulose membranes (Hybond, Amersham, UK) were coated with 25 µg of each spot-selected peptide and 1 µg of ZIKV-NS1, and for dot blot using human sera, the membranes were coated with 10 µg of each spot-selected peptide and 1 µg of ZIKV-NS1, using the Bio-Dot Apparatus (Bio-Rad, Hercules, CA, USA). The membranes were blocked with TBS supplemented with 5% of skim milk for 1 h at room temperature. After blocking, the membranes were washed with TBST 0.05% (TBS + 0.05% Tween 20 [*v*/*v*]). The mouse pAb anti-ZIKV-NS1 and human sera were diluted at 1:2000, and purified rabbit pAb anti-ZIKV-NS1 was diluted at 5 µg/mL in TBS supplemented with 2.5% skim milk. A protein G purified rabbit pAb anti-RBD protein of SARS-CoV-2 was used as a negative control at the same dilution. The mAb was diluted at 1 µg/mL in PBS supplemented with 2.5% skim milk. The membranes were then incubated for 2 h at room temperature. After this period, the membrane was washed again with TBST 0.05% and then incubated with HRP-labeled goat anti-rabbit IgG (1:5000, Santa Cruz Biotechnology™, Dallas, TX, USA), HRP-labeled goat anti-mouse IgG (1:5000; or 1:6000 for mAb, Santa Cruz Biotechnology™), or HRP labeled goat anti-human IgG (whole molecule, 1:5000, Sigma Aldrich), for 1 h at room temperature. The membranes were washed again with TBST 0.05%, and the reaction was revealed using SuperSignal™ West Pico PLUS Chemiluminescent Substrate (Thermo Fisher Scientific™, Waltham, MA, USA) following the manufacturer’s recommendations, and the images were recorded using iBright CL 1500 (Thermo Scientific™).

### 2.12. Peptide Titration Dot Blot Assay

The mAb reactivity against the ZKvROX1 was determined by checkerboard titration procedures. For dot blot assay, the Hybond membrane was coated with 20 µg, 15 µg, 10 µg, 5 µg, 2.5 µg, and 1.25 µg of spot-selected peptide 1 and 1 µg of ZIKV-NS1, respectively, using the Bio-Dot Apparatus (Bio-Rad). The membrane was blocked with TBS supplemented with 5% of skim milk for 1 h at room temperature. After blocking, the membrane was washed with TBST 0.05%. The mouse mAb was diluted at 1 µg/mL in TBS supplemented with 2.5% skim milk. The membrane was then incubated for 2 h at room temperature. After this period, the membrane was washed again with TBST 0.05% and then incubated with HRP-labeled goat anti-mouse IgG (1:8000, Santa Cruz Biotechnology™) for 1 h at room temperature. The membranes were washed again with TBST 0.05%, and the reaction was revealed using SuperSignal™ West Pico PLUS Chemiluminescent Substrate (Thermo Fisher Scientific™, Waltham, MA, USA) following the manufacturer’s recommendations, and the images were recorded using iBright CL 1500 (Thermo Fisher Scientific™, Waltham, MA, USA).

### 2.13. Indirect ELISA for ZIKV-NS1 Peptide Screening Specific IgG Antibody Detection

For ELISA, we used 18 serum samples, 9 for ZIKV-positive and 9 for ZIKV-negative patients. The optimum concentration of sera, peptides (ZKvROX1-ZKvROX6), and secondary antibodies, respectively, were determined by checkerboard titration procedures. Briefly, two high-binding 96-well plates MaxiSorp (Nunc^®^) were coated with 2 µg per well of each peptide, or a pool of peptides at 1 µg per well (0.167 µg of each peptide), diluted in carbonate/bicarbonate buffer at 4 °C overnight. The ZIKV-NS1 (1 µg per well) was used as a positive control. The next day, plates were washed three times with PBST 0.1% (PBS + 0.1% Tween 20 [*v*/*v*]). All washing steps were performed using an ELISA plate washer (Washwell plate, Robonik, Thane, India). Afterward, 100 µL per well of 3% skim milk diluted in PBST 0.1% was added to the plates and incubated for 1 h at room temperature as a blocking solution. After blocking, plates were washed six times with PBST 0.1% and incubated with 50 μL per well of human serum samples, diluted 1:50 in 1% skim milk solution for 1 h at room temperature. Next, plates were washed six times with PBST 0.1%, and then 50 µL of a 400 ng/µL dilution of goat anti-human IgG−HRP conjugated antibody diluted in 1% skim milk solution was added to wells, and the plates were incubated for 1 h at room temperature. Plates were washed again with PBST 0.1% and incubated for 15 min with 50 µL of One Step-TMB (3,3′,5,5′- tetramethylbenzidine) (Scienco Biotech). The reaction was stopped by the addition of 50 µL per well of 1 N sulfuric acid. The optical density at 450 nm (OD450) with a correction of 630 nm was measured using a Multiskan MS plate reader (Labsystems).

### 2.14. Evaluation and Optimization of the Synthetic Peptides in an ELISA Format

The optimum concentration of ZKvROX1 and ZKvROX4 peptides as coating antigens and secondary antibodies, respectively, were determined by checkerboard titration procedures. Briefly, two high-binding 96-well plates MaxiSorp (Nunc^®^) were coated with 1 µg/mL, 5 µg/mL, 10 µg/mL, or 20 µg/mL of ZKvROX1 or ZKvROX4, diluted in carbonate/bicarbonate buffer at 4 °C overnight. The next day, plates were washed three times with PBST 0.1% in the plate washer. Then, 100 µL per well of 3% skim milk diluted in PBST 0.1% was added to the plates and incubated for 1 h at room temperature as a blocking solution. After blocking, the plates were washed six times with PBST 0.1% and incubated with 50 µL per well of human serum samples, and diluted 1:50 in 1% skim milk solution for 1 h at room temperature. Next, the plates were washed six times with PBST 0.1%, and then 50 µL of a 1:500, 1:1000, 1:2000, or 1:4000 dilution of goat anti-human IgG−HRP conjugated antibody diluted in 1% skim milk solution was added to wells, and the plates were incubated for 1 h at room temperature. Plates were washed again with PBST 0.1% and incubated for 15 min with 50 µL per well of One Step-TMB (Scienco Biotech). The reaction was stopped by the addition of 50 µL per well of 1 N sulfuric acid. The optical density at 450 nm (OD450) with a correction of 630 nm was measured using a Multiskan MS plate reader (Labsystems).

### 2.15. Statistical Analyses

Statistically significant differences among human IgG levels and peptides in ELISA were determined by a Mann–Whitney test. *p* values < 0.05 were considered statistically significant. Statistical analyses and graphics were performed using GraphPad Prism v. 8.0.1 (GraphPad Software, San Diego, CA, USA).

## 3. Results

### 3.1. Immunization with ZIKV-NS1 Induced Specific Serum Antibody Responses in Rabbits and Mice

**Figure 1:**
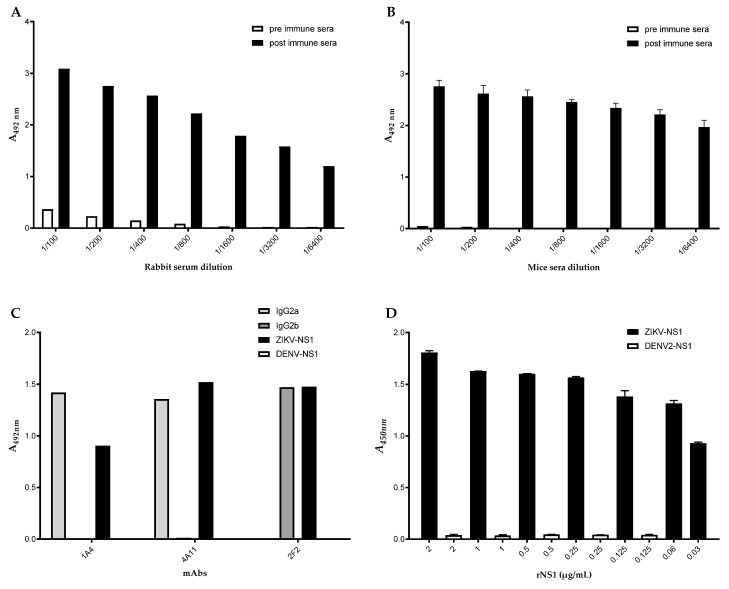
Detection of antigen-specific serum antibody responses in ZIKV-NS1 immunized animals. Microplates coated with ZIKV-NS1 were reacted with (**A**) rabbit or (**B**) mice serum samples diluted from 1/100 to 1/6400. (**C**) Reactivity of selected hybridomas by ELISA. Microplates coated with ZIKV-NS1 (black), DENV-NS1 (white), or different anti-IgG isotypes (IgG2a—light grey, IgG2b—dark grey) were reacted with hybridomas supernatant 1A4, 4A11, 2F2. (**D**) Captured ELISA reactivity of ZIKV-NS1. Microplates coated with rabbit pAb were reacted with ZIKV-NS1 (black columns) or DENV-NS1 (white columns) proteins at concentrations indicated in the figure. The reaction was captured after incubation with mAb 2F2.

In this step, we focused on the generation of hybridomas capable of producing specific anti-ZIKV-NS1 antibodies. Rabbits and mice were immunized with a two-dose regimen based on the ZIKV-NS1 antigen, a C-terminal fragment from ZIKV-NS1 protein that showed reduced serological cross-reactivity with anti-DENV serum samples (patent application number: BR 10 2016 0113180; [26,37]). As shown in Figure 1A,B, all immunized animals elicited anti-NS1 antibody responses with titers 1/6400 reaching OD higher than 1.0 and 2.0 in rabbits and mice, respectively (*p* < 0.05). As such, popliteal lymph node cells from ZIKV-NS1-immunized mice were fused with a non-Ig-secreting or synthesizing line derived from a cell line mouse myeloma P3 × 63Ag8 (SP2/O-Ag14). To avoid dengue cross-reaction, all hybridomas were selected by negative testing against DENV-NS1. A total of 12 secretory hybridomas were generated. Three of them presented ELISA with an OD higher than 1.0. Therefore, the clones were selected, subcloned by limiting dilution, and named 1A4, 4A11, and 2F2 (Figure 1C). The clones were expanded, supernatants collected, and mAbs purified for further characterization. Accordingly, mAbs 1A4 and 4A11 were characterized as IgG2a, and the 2F2 is an IgG2b secreting mAb.

The selected hybridomas were titrated by indirect ELISA, and the detection limits were 125 ng/mL, 60 ng/mL, and 15 ng/mL for 1A4, 4A11, and 2F2, respectively. Since the 2F2 mAb showed the lowest detection limit to ZIKV-NS1, we decided to check it by checkerboard titration with a rabbit pAb. By employing 15 μg/mL of capture antibodies and 2.5 μg/mL of pAb, the detection limit of the 2F2 mAb reached 3 ng/mL. For standardization, we first tested 61 sera samples, from which 27 and 34 were previously characterized as positive or negative for the presence of ZIKV, respectively. A total of 15/27 (55.5%) of the positive sera showed absorbance values higher than the cut-off, while 30/34 (88%) negative sera showed absorbance values below the cut-off. Further ELISA cross-reaction assurance was evaluated by the inclusion of dengue virus (DENV) and purified DENV-NS1 in the assays (Figure 1D).

### 3.2. The Common Consensus Motif Resulted in Clear Cross-Reactions with the NS1 Proteins of Other Flaviviruses

**Figure 2:**
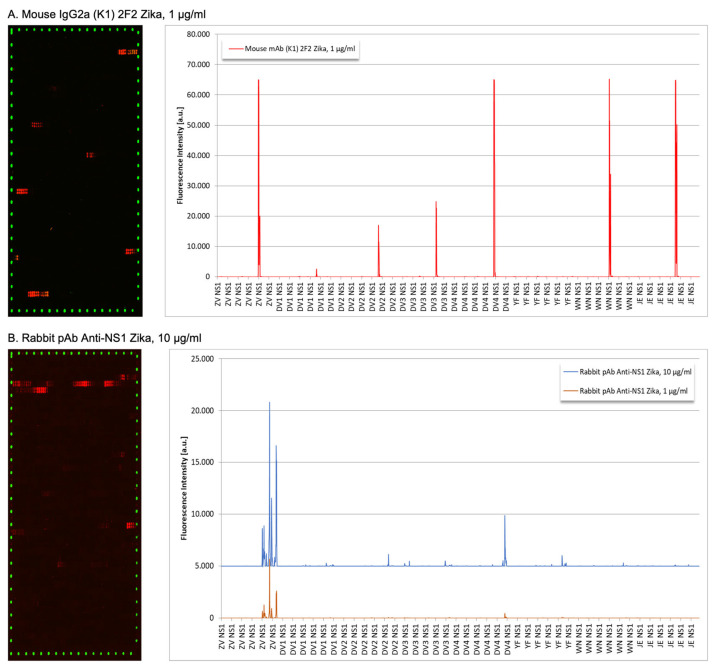
Peptide binding array profiles of anti-ZIKV NS1 mAb 2F2 and rabbit sera. (**A**) Mouse monoclonal 2F2 antibody; (**B**) rabbit polyclonal anti-ZIKV-NS1 sera were incubated in an NS1-based peptide microarray. The signal was captured after staining with secondary and control antibodies, as well as read-out at scanning intensities of 7/7 (red/green).

To localize the specific antibodies binding epitopes, peptide mapping array experiments were performed (Figure 2). The 2F2 ZIKV mAb exhibited weak to very strong antibody responses against epitope-like spot patterns formed by adjacent peptides with the consensus motifs REGYRT (ZIKV-NS1), QHNYRPGYFT (DENV1-NS1), QHNYRPGYHT (DENV2-NS1), SQHNYRPGYHT (DENV3-NS1), QHNYRQGYAT (DENV4-NS1), RRPGYKT (WNV-NS1) and REGYKT (JEV-NS1), with the common RxGYxT motif at high signal to noise ratios (Figure 2A) and (Supplementary File S1). Although the antibody was presumably raised against the NS1 protein of ZIKV, the common consensus motif resulted in clear cross-reactions with the NS1 proteins of DENV1, DENV2, DENV3, DENV4, WNV, and JEV. Only the NS1 protein of YFV with the consensus motif IPGYKV did not react with the ZIKV mouse monoclonal (2F2) IgG2a antibody.

Concerning rabbit polyclonal antibody IgG anti-ZIKV-NS1, it showed a moderate to strong polyclonal antibody response against epitope-like spot formed by adjacent peptides with the consensus motifs EGYRTQV, PWHSE, ECPGTKVYVEE, LRSTTASGR, CRECTMPPL and EPESNLVRSM (ZIKV NS1), SLRTTTASG (DENV2 NS1), SLRTTTVSG (DENV3 NS1), TMPPL (DENV4 NS1) and WCCRSCTMPPV (YFV NS1) at high signal-to-noise ratios (Figure 2B). We observed two common consensus motifs LR(S/T)TTxSG (ZIKV, DENV2, and DENV3) and TMPP(V/L) (ZIKV, DENV4, and YFV) as causes for the observed cross-reactions (Supplementary File S2).

**Figure 3:**
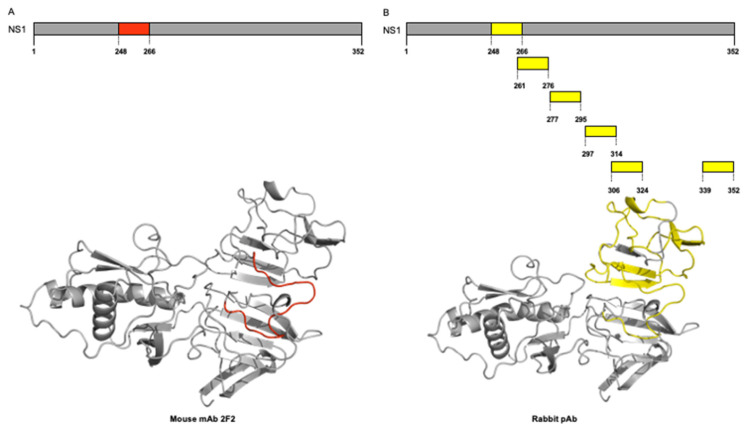
Three-dimensional visualization of identified epitopes of ZIKV-NS1 protein. Ribbon diagram representation. (**A**) NS1 protein is represented in grey, and three selected epitopes to the mAb 2F2 are colored red (position 248–266). (**B**) NS1 protein is represented in grey, and six selected epitopes to rabbit pAb are colored yellow (position 248–266, position 261–276, position 277–295, position 297–314, position 306–324, and position 339–352).

Finally, we performed three-dimensional visualization of the identified epitopes of the NS1 protein of ZIKV mapped for mAb 2F2 and pAb onto the corresponding three-dimensional structures on the crystal structure of ZIKV NS1 protein (PDB ID 5K6K). For this purpose, we used the ribbon diagram representation (Figure 3). We showed the NS1 protein, which is represented in grey, and one selected epitope colored red (position 248–266), representing the binding site of the 2F2 mAb (Figure 3A). As for the binding sites of the rabbit pAb, the NS1 protein is represented in grey, and six selected epitopes are colored yellow (positions 248–266, 261–276, 277–295, 297–314, 306–324, and 339–352) (Figure 3B). As expected, all mapped peptides are located at the C-terminal region of the ZIKV NS1 protein. Additionally, the cross-immunoreactive epitopes for ZIKV, DENV2, WNV, JEV, and YFV are shown for mAb 2F2 and pAb onto the corresponding three-dimensional structures, respectively (Supplementary File S2).

### 3.3. Peptides Generation by Mapping Arrays of ZIKV-NS1 Protein

**Figure 4:**
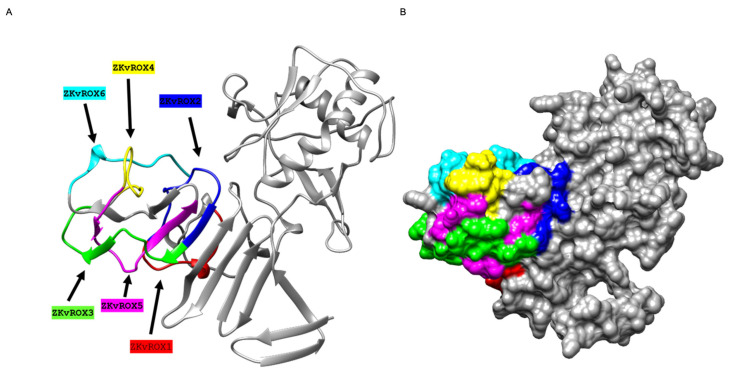
Three-dimensional visualization of selected epitopes of NS1 protein of ZIKV. (**A**) Ribbon diagram representation. NS1 protein is represented in grey, and six selected epitopes are colored red (ZKvROX1), blue (ZKvROX2), green (ZKvROX3), yellow (ZKvROX4), pink (ZKvROX5), and cyan (ZKvROX6). (**B**) The surface map of NS1 protein.

We mapped six peptides (Table 1) using mapping arrays from the ZIKV-NS1 protein sequence onto the corresponding three-dimensional structures on the crystal structure of ZIKV NS1 protein (PDB ID 5K6K). The six selected peptide sequences belong to the 246–351 motif of the NS1 protein, and the length of peptide sequences ranged from 13 to 19 amino acid residues (Figure 4 and Table 1).

### 3.4. In Silico Analyses Indicated High Antigenicity for Most Parts of the Selected Peptides

Selected epitope sequences were submitted to the NetNGly and NetPhos 3.1 software to predict N-glycosylation and phosphorylation sites (Table 2). Structural analysis showed that none of the identified epitopes present N-glycosylation sites. However, most of the selected peptides showed phosphorylation sites in their structure at serine residues, except for the ZKvROX3 peptide. The epitope ZKvROX4 showed three serine phosphorylation sites (Ser299, Ser302, Ser306). In addition, five of six selected peptide sequences of NS1 were predicted to have high antigenicity (the ABCPred score ranged from 0.65 to 0.87, and the VaxiJen score ranged from −0.27 to 0.95). The peptide ZKvROX6 showed the lowest antigenicity score propensity (ABCpre = 0.56 and VaxiJen = −0.2715).

Lee et al. (2018) [38] performed an in silico study with 10 flavivirus species (ZIKV, ILHV, JEV, SLEV, WNV, YFV, DENV1, DENV2, DENV3, DENV4) in which they identified sensitive and specific sequences of the E and NS1 proteins to differentiate the Zika virus from other arboviruses to improve antibody selection and diagnostic test specificity for this flavivirus. For that, the authors employed the sequences and tools available in Virus Pathogen Database and Analysis Resource-ViPR and Immune Epitope Database.

In order to compare the experimentally identified epitopes (ZKvROX1, ZKvROX2, ZKvROX3, ZKvROX4, ZKvROX5, and ZKvROX6) with the theoretical sequences by Lee et al. (2017) (116 from ZIKV; 70 from JEV; 57 from SELEV; 38 from WNV; 133 of YFV; 16 of DENV1; 23 of DENV2; 24 of DENV3; 33 of DENV4), the alignment was performed with the sequences from ZIKV (Supplementary File S4) or other arboviruses (Supplementary File S5). It was possible to observe, from the identity graph, highlighted in green, the complete homology in the GYRTQ sequence of the epitopes ZKvROX1 and ZKvROX2 and the KGPW segment with some theoretical epitopes indicated by Lee et al. (2018) [38] to identify NS1 from ZIKV. In the alignment between the experimental sequences of ZKvROX with the theoretical epitopes, the ZKvROX1 sequence (highlighted in red) was identified and showed the lowest homology among the other arboviruses (Supplementary File S5). The ZKvROX4 epitope presented identity among viruses and the second-highest immunogenicity score.

The peptides were also submitted to the Immune Epitope Database (IEDB) program (http://tools.iedb.org/main/, accessed on 25 October 2022) to generate theoretical immunogenicity scores for each one (Supplementary Table S1). Despite the peptide ZKvROX3 achieving the highest score, it had a low identity with the segments identified by Lee et al. (2018) [38] and low homology with the NS1 sequence from the other analyzed viruses. That result may be indicative of a specific sequence not previously described in the literature.

### 3.5. Validation of NS1 Protein-Derived Peptides Using Dot Blot Assays

**Figure 5:**
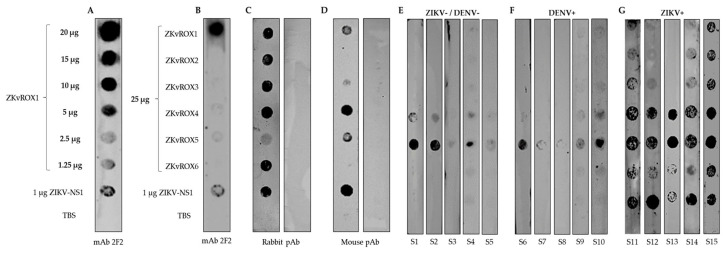
Reactivity of serum antibodies against the peptides (ZKvROX1-ZKvROX6) and ZIKV-NS1 by dot blot assays. (**A**) Serial dilution of ZKvROX1 against mAb 2F2. (**B**) Reactivity of all peptides (25 μg/mL) against the mAb 2F2. (**C**) Rabbit pAb immunized with the recombinant ZIKV-NS1 (left strip) or SARS-CoV-2 RBD protein as negative control (right strip). (**D**) Mouse immunized with DNA plasmid containing full ZIKV-NS1 (left strip) or mock group (right strip). (**E**) Human ZIKV and DENV negative sera (S1–S5). (**F**) Human sera from patients with DENV natural infections (S6–S10). (**G**) Human sera from patients with ZIKV natural infections (S11–S15).

We established dot blot assays based on the six selected peptides or the ZIKV-NS1 (Figure 5). The dot blot assays showed the reactivity against the NS1 derived peptides and ZIKV NS1 protein, mouse immunized with plasmidial DNA serum, and rabbit immunized with the recombinant ZIKV NS1 protein, in addition to ZIKV and DENV human natural infections sera (Figure 5). Dot blot assays showed a strong reactivity of mouse mAb 2F2 to ZKvROX1 and less intensity to NS1 protein (Figure 5A). In addition, the reactivity of mAb against the ZKvROX1 was evaluated in several peptide dilutions, showing a reaction until the dilution of 5 µg/mL (Figure 5B). Also, the rabbit pAb showed high reactivity against ZKvROX4 and ZIKV-NS1 and a slight reactivity against the ZKvROX1 and ZKvROX5, compared to the control group (protein G purified rabbit pAb anti-RBD (Receptor Binding Domain) protein of SARS-CoV-2) (Figure 5C). The plasmidial DNA immunization in mice was able to generate an immune response against the peptides ZKvROX1, ZKvROX2, ZKvROX3, ZKvROX4, and ZKvROX6, while the control group did not show any reactivity (Figure 5D).

When using human sera from healthy individuals with no previous infections for both ZIKV and DENV, there was reactivity only for peptides ZKvROX5 and ZKvROX4 (Figure 5E). There was a low reactivity against peptide ZKvROX5 and a slight reaction for ZKvROX4 using sera from patients with DENV natural infections (Figure 5F). Serum from patients positive for ZIKV natural infections had reactivity for all peptides, which the ZKvROX1, ZKvROX2, ZKvROX3, and ZKvROX6 were majority specific for only ZIKV. The peptides ZKvROX1, ZKvROX2, and ZKvROX3 fail to react only to serum S13. However, the peptide ZKvROX6 was recognized by all ZIKV serums as the NS1 control antigen (Figure 5G). The peptides ZKvROX4 and ZKvROX5 showed immunoreactivity against all serum tested. These results show that ZKvROX4 and ZKvROX5 demonstrate cross-reactivity between sera negative for ZIKV and DENV or positive for ZIKV. However, ZKvROX1, ZKvROX2, ZKvROX3, and ZKvROX6 showed promise to differentiate infections from ZIKV patients to non-infected or DENV-infected patients in dot blot assays to detect ZIKV-specific infections.

### 3.6. ELISA Assays

**Figure 6:**
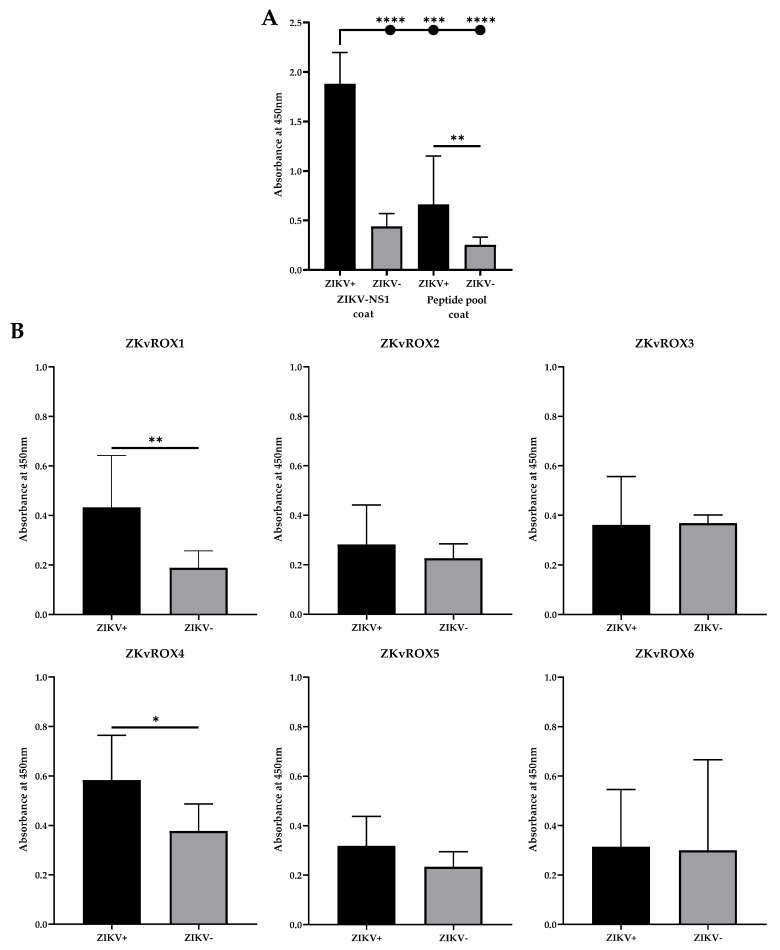
Reactivity of selected peptides by ELISA. (**A**) ELISA using the pool of the six peptides (P1–P6) and ZIKV-NS1 protein using serum from ZIKV infected patients or healthy individuals serum samples. (**B**) Reactivity of the peptides individually (ZKvROX1-ZKvROX6) tested by ELISA by using sera from infected or negative patients for ZIKV. Statistically significant differences among human IgG levels and peptides in ELISA were determined by a Mann–Whitney test. *p* values < 0.05 indicated statistically significant difference (* *p* < 0.05; ** *p* < 0.01; *** *p* < 0.001; **** *p* < 0.0001).

To evaluate the constructions, we used a pool containing equivalent amounts (0.167 µg) of each of the six peptides (ZKvROX1- ZKvROX6) as solid-phased antigens in ELISA assays. The results show that the peptide pool was able to differentiate sera from patients with natural ZIKV infections (*n* = 9) from healthy individual serum samples (*n* = 9) (*p* = 0.0053), besides the reaction signal was about 3 times lower for positive samples and about 2 times lower for negative samples when compared to the ZIKV-NS1 ELISA signal (Figure 6A). Still, further studies with more patient samples, including those with infections from other flaviviruses, especially DENV, are needed to investigate the effectiveness of such an approach.

When assessing the reactivity of the peptides individually, the ELISA results showed that when using sera positive or negative for ZIKV natural infections, ZKvROX1 and ZKvROX4 allowed statistically significant differences between positive and negative sera (*p* = 0.0070 and *p* = 0.0262, respectively), while the other peptides were not able to generate immunoreactivity (Figure 5C). Furthermore, ZKvROX1 and ZKvROX4 were the better ELISA-optimized peptides, showing the need for only 5 µg/mL of peptides and 400 ng/µL of conjugated antibodies for ideal performance and increased ratio between ZIKV+ and ZIKV- samples (Supplementary File S3).

## 4. Discussions

ZIKV and DENV share genetic and antigenic determinants [39]. This feature leads to difficulties in laboratory diagnosis based on immunoserological assays, particularly in endemic regions for these viruses. Indeed, the misdiagnoses of either ZIKV or DENV infections are caused by the cross-reactivity of antibodies from both infections [37].

One of the assays commonly employed for the early diagnosis of dengue is the InBios DENV Detect NS1 ELISA Kit. It has a reported sensitivity and specificity of 86.8% and 97.8%, respectively [40]; however, one potential issue may be the lack of sensitivity in secondary dengue cases, as reported in one study indicated 100% sensitivity in primary infections and 10% sensitivity in secondary infections [41]. Even so, the successful detection of DENV through NS1-based assays offers an attractive option for the development and optimization of immunoassays against other flaviviruses [42].

As such, ZIKV occurrence prompted the development of multiple antigen-capture assays, several of them based on the NS1 protein [43,44,45]. Some became commercially available for use in clinical diagnosis, while others were available for research use only. In fact, a portion of the developed diagnosis still remains untested in the field [45], while the tested ones showed sensitivities and specificities between 70% and 100% [46]. It is worth mentioning, though, that most of the serum panels used to validate such assays did not contain DENV and ZIKV serum samples obtained from DENV-endemic countries, thus lacking cross-reactive interference in the results.

Recent literature indicates that ZIKV serological tests based on C-terminal fragments of ZIKV non-structural protein 1 showed enhanced specificity [26,47]. Thus, in order to maximize the chances of avoiding cross-reaction in the present study, we employed a C-terminal fragment of ZIKV non-structural protein 1, previously reported to reduce the detection of cross-reactive anti-DENV antibodies [26,47] to generate polyclonal and mAbs. A capture assay was then designed and tested by checkerboard. The assays based on rabbit anti-ZIKV NS1 pAb and mAb 2F2 showed similar detection limits to DENV-NS1 capture ELISAs, such as SD Bioline’s detection limit of between 16 and 63 ng/mL NS1 [48]. Moreover, our results are in line with an antigen-capture ELISA configured with the affinity-purified anti-ZIKV NS1 pAb, for which the reported limit of detection was between 1.95 and 7.8 ng/mL [42].

The mentioned similarities guided us to test a small ZIKV sera panel obtained from infected patients and validated by qRT-PCR. The results showed an accuracy of 77.2%, which was superior to the one previously obtained by Beddingfield et al. (2021) [42], for which the detection limit was similar to the one obtained by us (55.5% vs. 55.7%). Additionally, other studies have been published with similar approaches based on capturing ZIKV-NS1 [45]. However, we could not compare them with our results due to the lack of tests with human clinical samples. Taken together, the data generated here appear accurate and can be led to a promising diagnostic test after improvement and evaluation against a broader and better-characterized ZIKV and DENV sera panel.

The second approach of the present study lies in the use of mAb 4F2 and the anti-ZIKV NS1 pAb sera to identify NS1 flavivirus epitopes by peptide mapping array. The array was composed of 15 aa linear peptides from ZIKV, DENV1, DENV2, DENV3, DENV4, YFV, WNV, and JEV NS1 proteins. For 2F2 mAb, the reactively identified epitopes were mostly conserved between ZIKV, DENV, WNV, and JEV in both linear and structural aspects. Previous studies have identified similar linear and discontinuous flavivirus epitopes from naturally infected humans [49]. The epitope REGYRT mapped on immunodominant region three of the NS1 loop has been previously described [42]. Through alignments of the NS1 full epitope sequence between flavivirus, there is a predominance of residues that are fully conserved, in addition to others, with a conservation between groups of strongly and weakly similar properties [50,51]. Checking the ZIKV, DENV1, DENV2, DENV3, DENV4, WNV, and JEV NS1 sequences, the common RxGYxT motif was possibly responsible for the cross-reactivity, highlighting the GY sequence conservation as critical amongst these flaviviruses [51]. On the other hand, this same IPGYKV region in YFV is not detected by the mAb 2F2, demonstrating that nearby sequences are essential for binding, as the YxT segment is described within the epitope of the mAb 22NS1 developed for JEV [50].

Surprisingly, the rabbit sera showed higher specificity for ZIKV epitopes through the peptide binding array. We believe the improved specificity of the pAbs in rabbit sera was conferred by the use of the C-terminal fragment of ZIKV-NS1 as the background for finding specific reactive epitopes [26,47]. We checked six distinct regions for highly reactive ZIKV, and only one region was detected with less intensity for DENV and YFV [49]. The ZIKV epitope EGYRTQV is located from immunodominant region three to the loop and from the epitopes LRSTTASGR (ZIKV) SLRTTTASG (DENV2), SLRTTTVSG (DENV3) to the immunodominant region four [42]. Moreover, the common motifs (LR(S/T)TTxSG and TMPP(V/L)) may be involved in cross-reactivity [52].

Antibody detection assays targeting NS1 have been extensively utilized for ZIKV infection, although expressive cross-reactivity still occurs [53]. Although all peptides played a role in the immune recognition of NS1 in humans, the peptides ZKvROX4 and ZKvROX6 showed greater specificity for ZIKV while peptide ZKvROX5 showed cross-reactivity against DENV human sera, according to dot-blot results. The ZIKV-NS1 recombinant protein tested here showed a strong ZIKV detection with reduced or no cross-reactivity with DENV-positive samples, supporting previous work [26,38,47]. In addition, three in silico predicted epitopes (ZKvROX3, ZKvROX5, and ZKvROX6) previously described by our group [54] were also tested. Interestingly, the epitope REGYRT identified with the 4F2 mAb is like region EGYRTQV of ZIKV-NS1 highlighted in the rabbit pAb mapping. Both peptides ZKvROX1 and ZKvROX4, which are linked to the immunodominant regions one and four of NS1, respectively, were able to detect antibodies by ELISA, confirming their immunodominance. Overall, these epitope regions were previously described by Lee et al. (2018) [38] within the peptides ZKvROX1, ZKvROX2, ZKvROX3, and ZKvROX6.

When analyzing the three-dimensional structure of ZIKV NS1, the six epitopes were arranged in the C terminal B ladder region (specifically in positions between 248–352) and showed pronounced exposure on the protein surface [42,55,56]. These data agree with the truncated ZIKV-NS1 (corresponding to the C terminal region) previously described and used to generate these antibodies [26,47]. From the conservation analysis of NS1 proteins among different flaviviruses, the most conserved surfaces are on the β-roll region and the C-terminal tip of the central β-ladder [51,57]. According to previous studies, the increased specificity of NS1 may be due mainly to the surface electrostatic potential of this region [16,51]. ZIKV-NS1 exhibits a variable surface and divergent electrostatic potential that may result in altered binding properties to host factors and protective antibodies distinct from the structures of other Flaviviruses [16,58].

In conclusion, we successfully generated high-quality antibodies capable of contributing to the development of antigen-capture diagnostic assays and carried out linear peptide arrays that indicate epitope sequences with the potential to contribute to the development of ZIKV-sensitive and specific serological tests. The peptides were chemically synthesized and detected the presence of ZIKV antibodies in ZIKV-infected convalescent sera. Although the study has some limitations, such as the identification of conformational epitopes and the limited number of convalescent serum samples, the results are promising and should be the starting point for the creation of new serological assays for the detection of ZIKV acute and convalescent phase infections.

## Figures and Tables

**Table 1:** Synthetic peptides from ZIKV-NS1 (ZIKASPPH2015—GenBank under the accession number KU321639).

NS1 Epitope	Residue	Peptide Length	Sequence Length
ZKvROX1	248–263	18	AGPLSHHNTREGYRTQVK
ZKvROX2	261–276	15	RTQVKGPWHSEELEI
ZKvROX3	277–295	18	RFEECPGTKVYVEETCGT
ZKvROX4	297–314	17	GPSLRSTTASGRVIEEW
ZKvROX5	306–324	19	VIEEWCCRECTMPPLSFRA
ZKvROX6	339–352	13	EPESNLVRSMVTA

**Table 2:** In silico analysis of selected epitopes of ZIKV-NS1 protein.

ZIKV NS1 Epitope	NetNGly	NetPhos 3.1	ABCpred	VaxiJen
ZKvROX1	None	Ser252	0.69	0.7651
ZKvROX2	None	Ser270	0.71	0.7841
ZKvROX3	None	None	0.87	0.1894
ZKvROX4	None	Ser299, Ser302, Ser306	0.65	0.4459
ZKvROX5	None	Ser321	0.69	0.9463
ZKvROX6	None	Ser342	0.56	−0.2715

## Data Availability

Correspondence and requests for materials should be addressed to C.R.P., D.L., or R.M.F.P.

## Supplementary Materials

The following supporting information can be downloaded at: https://www.mdpi.com/article/10.3390/v15030654/s1, File S1: General representation of the specifics of selected epitopes from linear epitope mapping; File S2: Topology of cross-immunoreactivity epitopes in the three-dimensional structure of NS1 protein for mouse mAb and rabbit pAb; File S3: Reactivity of peptides ZKvROX1 and ZKvROX4 by four dilutions of peptides between ZIKV+ and ZIKV- serum; File S4: Multiple alignment between theoretical ZIKV and identified experimental sequences; File S5: Multiple alignment between theoretical arbovirus and experimental ZIKV sequences; Table S1: Immunogenicity score of experimental ZIKV peptides.
