# Supplementary material for: Identification of Zika Virus NS1-Derived Peptides with Potential Applications in Serological Tests
Source: Viruses. 2023 Feb 28;15(3):654. doi: 10.3390/v15030654 (PMC10052002; doi:10.3390/v15030654)
Supplement: Supplementary file 1 [file viruses-15-00654-s001.zip › Legends of supplemental figures.pdf]

Supplementary Figures

Figure S1

General representation of the specifics of selected epitopes from linear epitope mapping. **A)** Set of identified epitopes for mouse and rabbit antibodies. **B)** Multiple alignment performed by Clustal showing the conserved sequences between the NS1 from DV, JE, WN and ZV for mouse and YF, DV and ZV. **C)** Phylogenetic analysis to represent the similarity between the epitopes from flavivirus NS1 identified (DV, JE, WN and ZV for mouse and YF, DV and ZV). **D)** The consensus sequences RxGYxT and LR(S/T)TTxSG are represented in the form of a logo.

Figure S2

Topology of cross-immunoreactivity epitopes in the three-dimensional structure of NS1 protein for mouse mAb and rabbit pAb. In Figure S2A, the common epitope for ZIKV, DENV, WNV and JEV is shown in red color, respectively. In figure S2B, the common epitopes for ZIKV, DENV and YFV are shown in yellow color, respectively.

Figure S3. Reactivity of peptides ZKvROX1 and ZKvROX4 by four dilutions of peptides between ZIKV+ and ZIKV- serum IgG antibodies.

Figure S4.

Multiple alignment between theoretical ZIKV and identified experimental sequences. The graph represents the alignment of amino acids: Green 100% identity; Yellow at least 30% and under 100% identity; Red below 30% identity. Residues highlighted in black show 100% identity. In lead gray 80% to 100% identity. In light gray from 60% to 80% identity. Blank identity less than 60%.

Figure S5.

Multiple alignment between theoretical arbovirus and experimental ZIKV sequences. The graph represents the alignment of amino acids: Green 100% identity; Yellow at least 30% and under 100% identity; Red below 30% identity. Homology scale between the residues highlighted in the graph: black 100% homology; Lead gray 80% to 100%; Light gray from 60% to 80%; White less than 60%.
