# Supplementary material for: Identification of Zika Virus NS1-Derived Peptides with Potential Applications in Serological Tests
Source: Viruses. 2023 Feb 28;15(3):654. doi: 10.3390/v15030654 (PMC10052002; doi:10.3390/v15030654)
Supplement: Supplementary file 1 [file viruses-15-00654-s001.zip › Supplementary File S1.pdf]

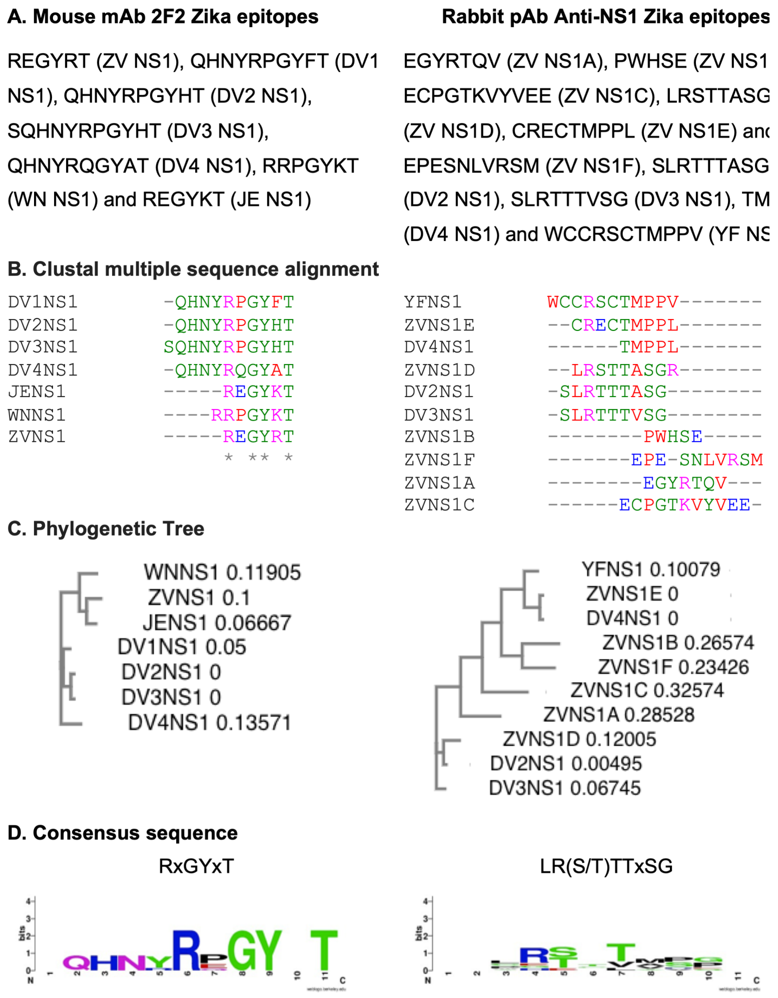

Figure S1. General representation of the specifics of selected epitopes from linear epitope mapping. A) Set of identified epitopes for mouse and rabbit antibodies. **B)** Multiple alignments performed by Clustal showing the conserved sequences between the NS1 from DV, JE, WN and ZV for mouse and YF, DV and ZV. **C)** Phylogenetic analysis to represent the similarity between the epitopes from flavivirus NS1 identified (DV, JE, WN and ZV for mouse and YF, DV and ZV). **D)** The consensus sequences RxGYxT and LR(S/T)TTxSG are represented in the form of a logo.
