# Supplementary material for: Identification of Zika Virus NS1-Derived Peptides with Potential Applications in Serological Tests
Source: Viruses. 2023 Feb 28;15(3):654. doi: 10.3390/v15030654 (PMC10052002; doi:10.3390/v15030654)
Supplement: Supplementary file 1 [file viruses-15-00654-s001.zip › Supplementary File S2.pdf]

A. Mouse mAb (K1) 2F2 ZIKV

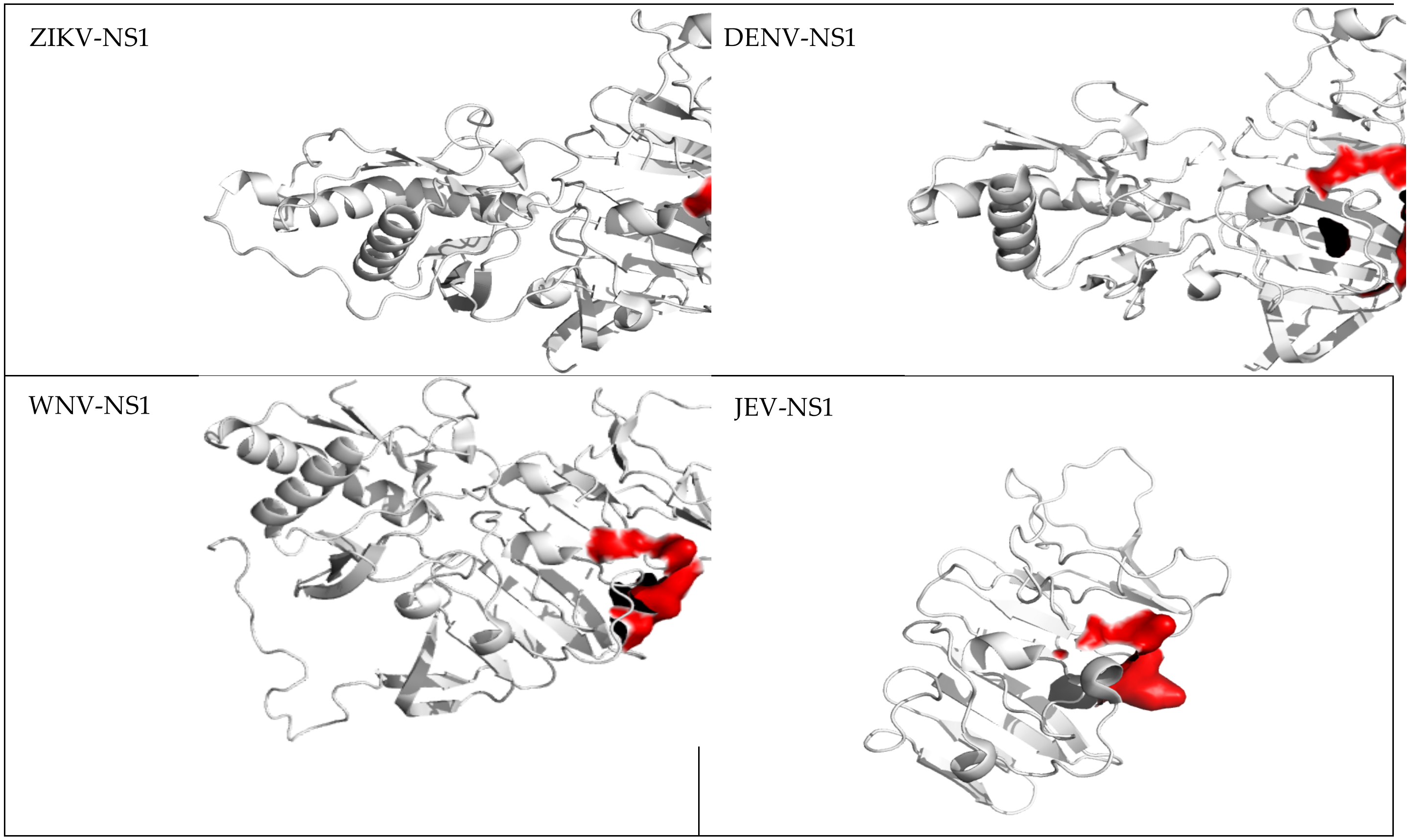

B. Rabbit pAb Anti-NS1 ZIKV

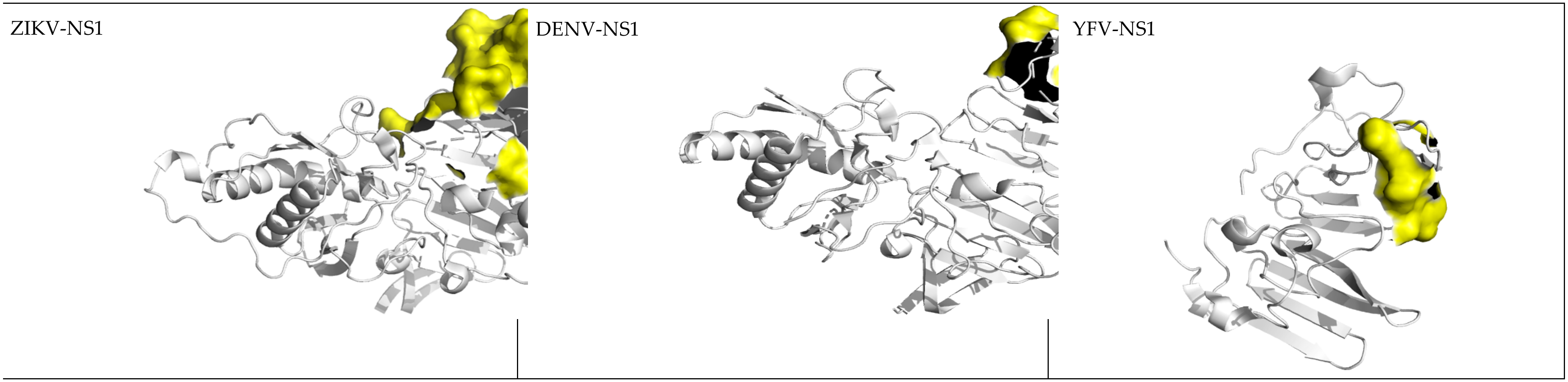

Figure S2. Topology of cross-immunoreactivity epitopes in the three-dimensional structure of NS1 protein for mouse mAb and rabbit pAb. In Figure S2A, the common epitope for ZIKV, DENV, WNV and JEV is shown in red color, respectively. In figure S2B, the common epitopes for ZIKV, DENV and YFV are shown in yellow color, respectively
