# Supplementary material for: Identification of Zika Virus NS1-Derived Peptides with Potential Applications in Serological Tests
Source: Viruses. 2023 Feb 28;15(3):654. doi: 10.3390/v15030654 (PMC10052002; doi:10.3390/v15030654)
Supplement: Supplementary file 1 [file viruses-15-00654-s001.zip › Supplementary File S4.pdf]

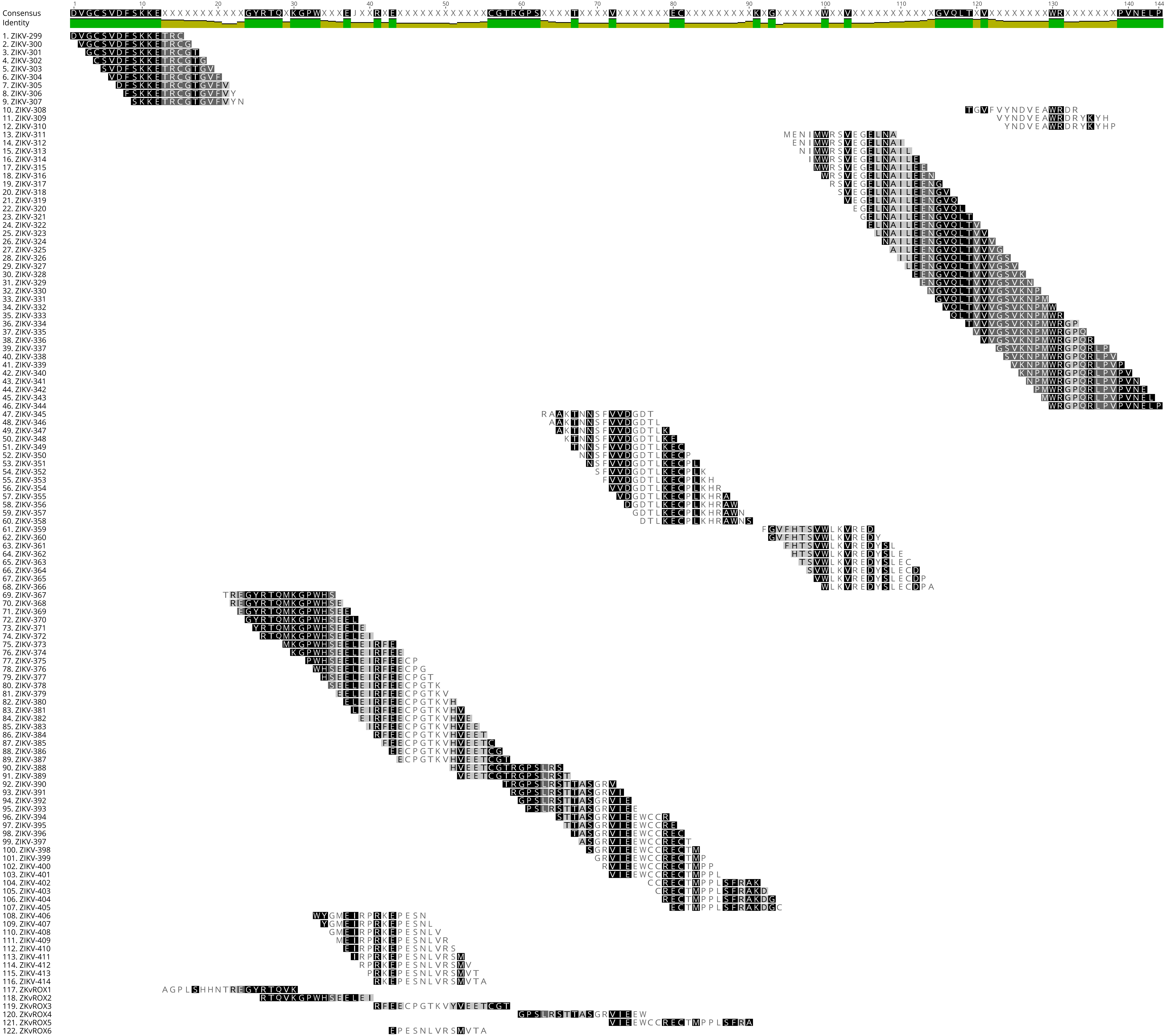

1 10 20 30 40 50 60 70 80 90 100 110 120 130 140 144
Consensus DVGCSVDFSKKEXXXXXXXXXXXGYRTQXKGPWXXXEJXXRXEXXXXXXXXXXXXCGTRGPSXXXXTXXXXVXXXXXXXECXXXXXXXXXXKXGXXXXXXWXXXVXXXXXXXXXXXGVQLTXVXXXXXXXXWRXXXXXXXPVNELP
Identity
1. ZIKV-299 DVGCSVDFSKKETRC
2. ZIKV-300 VGCSVDFSKKETRCG
3. ZIKV-301 GCSVDFSKKETRCGT
4. ZIKV-302 CSVDFSKKETRCGTG
5. ZIKV-303 SVDFSKKETRCGTGV
6. ZIKV-304 VDFSKKETRCGTGVF
7. ZIKV-305 DFSKKETRCGTGVFV
8. ZIKV-306 FSKKETRCGTGVFVY
9. ZIKV-307 SKKETRCGTGVFVYN
10. ZIKV-308 TGVFVYNDVEAWRDR
11. ZIKV-309 VYNDVEAWRDRYKYH
12. ZIKV-310 YNDVEAWRDRYKYHP
13. ZIKV-311 MENIMWRSVEGELNA
14. ZIKV-312 ENIMWRSVEGELNAI
15. ZIKV-313 NIMWRSVEGELNAIL
16. ZIKV-314 IMWRSVEGELNAILE
17. ZIKV-315 MWRSVEGELNAILEE
18. ZIKV-316 WRSVEGELNAILEEN
19. ZIKV-317 RSVEGELNAILEENG
20. ZIKV-318 SVEGELNAILEENGV
21. ZIKV-319 VEGELNAILEENGVQ
22. ZIKV-320 EGELNAILEENGVQL
23. ZIKV-321 GELNAILEENGVQLT
24. ZIKV-322 ELNAILEENGVQLTV
25. ZIKV-323 LNAILEENGVQLTVV
26. ZIKV-324 NAILEENGVQLTVVV
27. ZIKV-325 AILEENGVQLTVVVG
28. ZIKV-326 ILEENGVQLTVVVGS
29. ZIKV-327 LEENGVQLTVVVGSV
30. ZIKV-328 EENGVQLTVVVGSVK
31. ZIKV-329 ENGVQLTVVVGSVKN
32. ZIKV-330 NGVQLTVVVGSVKNP
33. ZIKV-331 GVQLTVVVGSVKNPM
34. ZIKV-332 VQLTVVVGSVKNPMW
35. ZIKV-333 QLTVVVGSVKNPMWR
36. ZIKV-334 TVVVGSVKNPMWRGP
37. ZIKV-335 VVVGSVKNPMWRGPQ
38. ZIKV-336 VVGSVKNPMWRGPQR
39. ZIKV-337 GSVKNPMWRGPQRLP
40. ZIKV-338 SVKNPMWRGPQRLPV
41. ZIKV-339 VKNPMWRGPQRLPVP
42. ZIKV-340 KNPMWRGPQRLPVPV
43. ZIKV-341 NPMWRGPQRLPVPVN
44. ZIKV-342 PMWRGPQRLPVPVNE
45. ZIKV-343 MWRGPQRLPVPVNEL
46. ZIKV-344 WRGPQRLPVPVNELP
47. ZIKV-345 RAAKTNNSFVVDGDT
48. ZIKV-346 AAKTNNSFVVDGDTL
49. ZIKV-347 AKTNNSFVVDGDTLK
50. ZIKV-348 KTNNSFVVDGDTLKE
51. ZIKV-349 TNNSFVVDGDTLKEC
52. ZIKV-350 NNSFVVDGDTLKECP
53. ZIKV-351 NSFVVDGDTLKECPL
54. ZIKV-352 SFVVDGDTLKECPLK
55. ZIKV-353 FVVDGDTLKECPLKH
56. ZIKV-354 VVDGDTLKECPLKHR
57. ZIKV-355 VDGDTLKECPLKHRA
58. ZIKV-356 DGDTLKECPLKHRAW
59. ZIKV-357 GDTLKECPLKHRAWN
60. ZIKV-358 DTLKECPLKHRAWNS
61. ZIKV-359 FGVFHTSVWLKVRED
62. ZIKV-360 GVFHTSVWLKVREDY
63. ZIKV-361 FHTSVWLKVREDYSL
64. ZIKV-362 HTSVWLKVREDYSLE
65. ZIKV-363 TSVWLKVREDYSLEC
66. ZIKV-364 SVWLKVREDYSLECD
67. ZIKV-365 VWLKVREDYSLECDP
68. ZIKV-366 WLKVREDYSLECDPA
69. ZIKV-367 TREGYRTQMKGPWHS
70. ZIKV-368 REGYRTQMKGPWHSE
71. ZIKV-369 EGYRTQMKGPWHSEE
72. ZIKV-370 GYRTQMKGPWHSEEL
73. ZIKV-371 YRTQMKGPWHSEELE
74. ZIKV-372 RTQMKGPWHSEELEI
75. ZIKV-373 MKGPWHSEELEIRFE
76. ZIKV-374 KGPWHSEELEIRFEE
77. ZIKV-375 PWHSEELEIRFEECP
78. ZIKV-376 WHSEELEIRFEECPG
79. ZIKV-377 HSEELEIRFEECPGT
80. ZIKV-378 SEELEIRFEECPGTK
81. ZIKV-379 EELEIRFEECPGTKV
82. ZIKV-380 ELEIRFEECPGTKVH
83. ZIKV-381 LEIRFEECPGTKVHV
84. ZIKV-382 EIRFEECPGTKVHVE
85. ZIKV-383 IRFEECPGTKVHVEE
86. ZIKV-384 RFEECPGTKVHVEET
87. ZIKV-385 FEECPGTKVHVEETC
88. ZIKV-386 EECPGTKVHVEETCG
89. ZIKV-387 ECPGTKVHVEETCGT
90. ZIKV-388 HVEETCGTRGPSLRS
91. ZIKV-389 VEETCGTRGPSLRST
92. ZIKV-390 TRGPSLRSTTASGRV
93. ZIKV-391 RGPSLRSTTASGRVI
94. ZIKV-392 GPSLRSTTASGRVIE
95. ZIKV-393 PSLRSTTASGRVIEE
96. ZIKV-394 STTASGRVIEEWCCR
97. ZIKV-395 TTASGRVIEEWCCRE
98. ZIKV-396 TASGRVIEEWCCREC
99. ZIKV-397 ASGRVIEEWCCRECT
100. ZIKV-398 SGRVIEEWCCRECTM
101. ZIKV-399 GRVIEEWCCRECTMP
102. ZIKV-400 RVIEEWCCRECTMPP
103. ZIKV-401 VIEEWCCRECTMPPL
104. ZIKV-402 CCRECTMPPLSFRAK
105. ZIKV-403 CRECTMPPLSFRAKD
106. ZIKV-404 RECTMPPLSFRAKDG
107. ZIKV-405 ECTMPPLSFRAKDGC
108. ZIKV-406 WYGMEIRPRKEPESN
109. ZIKV-407 YGMEIRPRKEPESNL
110. ZIKV-408 GMEIRPRKEPESNLV
111. ZIKV-409 MEIRPRKEPESNLVR
112. ZIKV-410 EIRPRKEPESNLVRS
113. ZIKV-411 IRPRKEPESNLVRSM
114. ZIKV-412 RPRKEPESNLVRSMV
115. ZIKV-413 PRKEPESNLVRSMVT
116. ZIKV-414 RKEPESNLVRSMVTA
117. ZKvROX1 AGPLSHHNTREGYRTQVK
118. ZKvROX2 RTQVKGPWHSEELEI
119. ZKvROX3 RFEECPGTKVYVEETCGT
120. ZKvROX4 GPSLRSTTASGRVIEEW
121. ZKvROX5 VIEEWCCRECTMPPLSFRA
122. ZKvROX6 EPESNLVRSMVTA
