# Supplementary material for: Identification of Zika Virus NS1-Derived Peptides with Potential Applications in Serological Tests
Source: Viruses. 2023 Feb 28;15(3):654. doi: 10.3390/v15030654 (PMC10052002; doi:10.3390/v15030654)
Supplement: Supplementary file 1 [file viruses-15-00654-s001.zip › Supplementary Table S1.pdf]

Table S1. Immunogenicity score of experimental ZIKV peptides.

Name	Peptide	Length	Score
ZKvROX1	AGPLSHHNTREGYRTQVK	18	0,10924
ZKvROX2	RTQVKGPWHSEELEI	15	0,14313
ZKvROX3	RFEECPGTKVYVEETCGT	18	0,21339
ZKvROX4	GPSLRSTTASGRVIEEW	17	0,16892
ZKvROX5	VIEEWCCRECTMPPLSFRA	19	0,14706
ZKvROX6	EPESNLVRSMVTA	13	-0,32935
