# Supplementary figures and images for: Identification of Zika Virus NS1-Derived Peptides with Potential Applications in Serological Tests
Source: Viruses. 2023 Feb 28;15(3):654. doi: 10.3390/v15030654 (PMC10052002; doi:10.3390/v15030654)

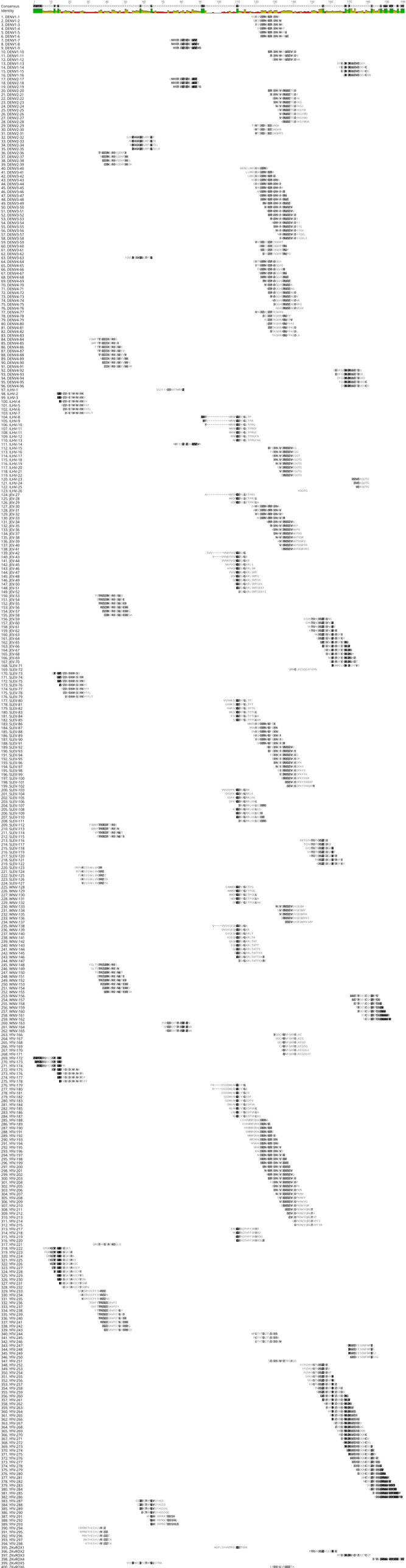

Supplement: Supplementary file 1 [file viruses-15-00654-s001.zip › Supplementary File S5.pdf]
